# Digital technology, emotions, and social relationships in dementia care: A narrative review

**DOI:** 10.1177/20552076251411208

**Published:** 2026-01-21

**Authors:** Marcus Persson, Ann-Charlotte Bivall, Elin Thunman

**Affiliations:** 1 4566 Department of Education and Sociology, Institution of Behavioral Sciences and Learning, Linköping University, Linköping, Sweden; 2 8097 Department of Sociology, Uppsala University, Uppsala, Sweden

**Keywords:** Digital technology, dementia care, social bonds, emotions, interaction, affordances

## Abstract

The aim of this review is to explore how the (dis)affordances of digital technologies—such as robots, game consoles, tablet computers, and virtual environments—shape the quality of the social relationships between care workers and residents in dementia care. Using a narrative review methodology, we analyzed 15 peer-reviewed articles published between 2012 and 2023 through thematic analysis. The findings are organized around three contrasting emotional dimensions: comfort–discomfort, joy–anger, and safety–vulnerability. Comfort and discomfort were primarily linked to everyday conversations, joy and anger to experiences of play and entertainment, and safety and vulnerability to how effectively care workers safeguarded residents’ sense of security. These results demonstrate that emotional expressions are not only outcomes of interpersonal interaction but are also mediated by technological affordances. Supporting care workers in recognizing and responding to these emotional dynamics is therefore crucial for ensuring that digital technologies enhance residents’ well-being and help sustain secure social bonds in dementia care.

## Introduction

The adoption of digital technology in elderly care—and residential dementia care in particular—has garnered significant research attention in recent years. Technologies designed to assist the elderly in various ways are generally seen as enhancing the quality and efficiency of care services.^1^ Examples include the use of assistive and social robots,^2^ sensors, cameras, and automatic alarms,^3^ tablets with various virtual applications,^4^ as well as virtual experience technologies and digital games.^5^ Existing studies have revealed diverse outcomes concerning the attitudes and behaviors of both care workers and dementia care residents toward such digital aids.^6,7^ For example, some studies indicate that social robots and tablets in care settings can facilitate communication and embodied interaction between care workers and residents, emphasizing the social and physical aspects of their interactions.^8,9^ However, there are also studies that observe ethical dilemmas, for instance, if assistive technology serves to replace the care worker and leave the resident alone without supervision for longer periods.^10,11^

Despite mixed results, there is a growing consensus among researchers that the outcomes of digital technology in care must be understood within the framework of “triadic interaction”^12^ between care workers, residents, and technology. From a sociotechnical perspective, technology functions as an agential object embedded in care practices.^13,14^ For instance, care workers may need to facilitate conversations between residents and robots,^15^ ensure that the technology is accessible at the appropriate moment,^16^ and “stage” it by handling it in a particular way, discussing its functions, and sometimes physically guiding users in its operation.^17–19^

By acknowledging both human and technological agency, we examine the social relationship between care workers and residents, within which technology plays a mediated role.^20,21^ Taking the triadic interaction between care workers, residents, and technology as a starting point, this review undertakes the novel approach of studying emotions as indicators of the quality of the social relationship between care workers and dementia care residents in their encounters with digital technology.

Drawing upon the American sociologist Thomas Scheff's theory about social bonds, we will examine the emotional expressions of both care workers and residents when interacting with the help of digital technology. Scheff's theory builds on the assumption that there is “a crucial connection between the state of the bond and emotions, how shame signals the state of the bond, and how unacknowledged shame explosively disrupts the bond.”^22^^(p72)^ We adopt this perspective to understand emotional expressions as indicators of the quality of the relationship between different actors.

While Scheff's theory has informed research in contexts like education—where secure or insecure bonds affect learning outcomes^23,24^—it does not account for the role of technology in shaping human interaction. To address this, we integrate affordance theory, which highlights the reciprocal relationship between individuals and their material environments. Specifically, we use the concept of (dis)affordances to explore how digital technologies mediate social engagement and evoke emotional expression between care workers and residents.

By reviewing the existing research on digital technology in dementia care, the aim of this paper is to explore the impact of the (dis)affordances of digital technology on the social bond (in other words, the quality of the relationship) between care workers and residents in a dementia care context. Three research questions will guide the study:

- Which emotional expressions are identified when care workers and residents engage with different digital technologies in their interactions?
- What do the emotional expressions indicate about the quality of the social bond between care workers and residents?
- How do the (dis)affordances of different digital technologies reinforce or challenge the social bonds between care workers and residents?

By deepening our understanding of how care technologies shape emotional and social dynamics, this research lays the groundwork for more responsive and person-centered dementia care. It highlights the importance of designing and implementing technologies that are sensitive to the relational and emotional needs of residents and care workers alike.

The review begins with a presentation of the theoretical framework consisting of Scheff's theory of social bonds in combination with the theory of (dis)affordances. Thereafter, we describe the methodological approach of the review and the workflow process. Adopting a thematic approach, the findings account for the main emotion categories identified in the reviewed papers: comfort–discomfort, joy–anger, and safety–vulnerability. At the end of the paper, we discuss how an emotional analytical approach can help to better understand how user engagement with digital technology in dementia care influences the relationships between care workers and residents.

## Theory: social bonds and technological (dis)affordances

In dementia care research, emotions have typically been approached through a biomedical lens, where affective expressions such as agitation or anxiety are interpreted as symptoms of underlying cognitive decline.^2^ Within this paradigm, digital technologies such as social robots are typically evaluated for their ability to mitigate these symptoms, offering calming or distracting effects for residents.^15,16^ While these findings are important, they tend to frame emotions as problems to be solved, rather than as meaningful signals embedded within social relationships as in a biopsychosocial approach to dementia care.^25,26^

In this study, we adopt a relational perspective to understand emotions as part of a “triadic relationship” between care workers, residents, and technological objects.^12,17^ Drawing on Thomas Scheff's theory of social bonds in conjunction with the theory of (dis)affordances,^27,28^ we conceptualize emotional expression not as isolated individual states, but as indicators of the quality of technology-mediated social relations. Our theoretical standpoint is, thus, that emotions are created and displayed in the social relationships between dementia care workers and residents in their encounters with digital technology.

At the core of Scheff's theory lies the assumption that human beings are fundamentally social and have an intrinsic need for emotional connection and recognition.^22,29,30^ Influenced by the interactionist traditions of Cooley^31^ and Goffman,^32^ Scheff argues that emotions arise from our perception of how others view us. A person's sense of pride or shame arises in response to whether they feel acknowledged, respected, or devalued in the eyes of others. These feelings are not trivial; they are foundational to the regulation and maintenance of what Scheff terms *social bonds*—dynamic, provisional ties between individuals that are continually renegotiated through interaction.

Scheff conceptualizes these bonds as being either secure or fragile. Secure social bonds are characterized by emotional attunement and “substantial mutual understanding of each other's thoughts, beliefs, and feelings. That is, a secure bond exists when two persons accurately understand each other's interior life.”^22^^(p65)^ Fragile bonds, on the other hand, are characterized by emotional incoherence, misunderstanding, or disrespect. Emotions function as a barometer of these bonds: pride and related feelings (e.g., joy, comfort, satisfaction) suggest strong relational bonds, whereas shame (broadly defined to include shyness, embarrassment, fear, or anger) indicates threats to those bonds.^30^

Despite its strengths, Scheff’s theory is primarily interpersonal and does not address the role of technology in the triadic interaction between care worker, resident, and technological object. To investigate how digital technologies shape modes of engagement within social contexts, we draw on the concept of (dis)affordances—possibilities or constraints for action that arise from the relational dynamics between actors and their environments.

The theory of affordances stems from ecological psychology^27,28^ and further develops through its sociological extensions in science and technology studies.^33,34^ Affordances refer to the possibilities for action that arise from the interaction between an actor and their environment. Importantly, these possibilities are not fixed properties of technological objects but are emergent, situated, and contingent on the user's capacities, perceptions, and emotional dispositions.^35^

Affordances, then, are deeply relational: what technology affords a user depends on the social context, the history of interaction, and the emotional texture of that moment. In this light, technologies do not simply “function” in objective ways—they mediate relationships, shape expectations, and elicit or suppress emotions. Moreover, some design features encode disaffordances, which actively preclude access or engagement for certain users.^36^ These disaffordances may not only inhibit practical use but also erode users’ sense of dignity, agency, or belonging, potentially weakening social relations and causing interpersonal disengagement or abandonment.^37^

By synthesizing Scheff's theory of social bonds with the theory of (dis)affordances, we offer an integrated framework for understanding the socioemotional dynamics between care workers and residents when engaging with digital technologies. These technologies are not just functional tools; they are relational artifacts that contribute to the ongoing construction, maintenance, or degradation of emotional bonds. Technologies afford not only certain actions but also specific kinds of feelings—facilitating pride and mutual recognition in some cases or exacerbating shame and social disconnection in others.

This perspective has relevance in dementia care, where verbal communication and cognitive coherence may be compromised. As residents increasingly rely on affective cues to express and interpret social intent, emotions become a primary medium for negotiating social bonds. For care workers, this places a premium on “relational competence”^23^—the capacity to recognize, interpret, and manage not only the emotions of residents, but also their own emotions, especially in moments mediated by digital technologies. Understanding how technologies offer or constrain this emotional labor is therefore critical to evaluating their role in care.

In sum, we argue that an integrated approach offers a richer understanding of technologically mediated care. It allows us to move beyond instrumental notions of “what technology does,” toward a deeper inquiry into how technologies participate in the making and unmaking of social bonds and caring relations.

## Method: narrative review with thematic analysis

Unlike systematic reviews, which typically address a narrow research question with prespecified methods to synthesize comparable studies, narrative reviews offer greater flexibility. They can draw on a wide range of studies and provide not only a summary of existing knowledge, but also interpretation and critique.^1,38^ Such an approach is particularly valuable where knowledge is limited, where new theoretical perspectives are emerging, or where current evidence benefits from being viewed through an alternative lens. In our case, the contribution lies in adopting an emotional approach to interpret studies on the adoption of digital technologies in dementia care. We also did not adopt a scoping review method, as our aim was not to map the breadth of the literature but to engage in critical interpretation. A narrative review was therefore more suitable, and to address common critiques of this approach,^38^ we applied rigorous methods in our literature search, inclusion and exclusion criteria, and quality control.

To ensure both breadth and depth of understanding, the study first employed a narrative review search before proceeding to thematic analysis. Conducting a narrative review at the outset allowed for the identification and synthesis of a broad range of relevant literature, mapping out the main areas of focus within the field. Building on these insights, a thematic analysis was then undertaken to systematically examine and interpret patterns across the literature. In line with the aim of the study, we developed an account of how emotional expressions—mediated by technological affordances—shape social bonds in dementia care. This combination of narrative review and thematic analysis thus enabled us to capture both the scope of existing knowledge and its underlying meanings.

### Search strategy

The work began by defining the content, focus, and limits of the study according to the project's aim and research questions. We collaborated with a group of librarians at Linköping University to organize the search using a method that is often used for defining a clear and relevant search, the PCC (Population, Context, Concept) framework.^39^ From a preliminary search in the PubMed database and an initial assessment of pertinent abstracts, a set of search terms was gradually developed. This led to the inclusion of further search terms. While the full search string is reproduced as an Appendix, the short version (corresponding to PCC) is: Dementia* (population) AND Health personnel* AND Social interaction (context a) AND digital technology* (context b) AND emotions* (concept).

### Databases and time frame

Searches were conducted between September and October 2023 in five electronic databases: PubMed, CINAHL, PsycINFO, Scopus, and Web of Science. The time frame was limited to publications from 2012 to 2023, reflecting the period in which digital technologies began to be integrated into dementia care practice.

### Inclusion and exclusion criteria

Only original peer-reviewed and empirically based articles in English published in academic journals were included. This meant that review papers and theoretical or conceptual papers were excluded.

A further criterion was the focus on the subject of technology-mediated interactions between care workers and residents in dementia care. Papers that dealt with only one of the actors (be it care worker or resident) were excluded. Papers which focused on homecare relationships between informal caregivers (such as spouses or relatives) and residents were also excluded. Similar arguments led us to exclude papers that focused on care students’ perspectives on digital technology.

As explained above, we treat emotions as an indicator of the quality of the relationship between care worker and resident. As such, emotions were a crucial criterion in the search. We found several papers that dealt with emotions as symptoms of dementia and other degenerative disorders. These papers were excluded due to their biomedical focus. We only included papers that dealt with the relational aspects of emotions, that is, with emotional reactions to digital technology in the interactions between care worker and resident.

We deliberately adopted a broad definition of digital technology and sought to be as inclusive as possible. The only inclusion criterion was that technology had to have a mediating role or form some part of the interaction between care workers and residents. This led us to exclude articles discussing technologies which did not involve any social interaction, such as surveillance technology.

### Screening and selection process

**Figure 1.**
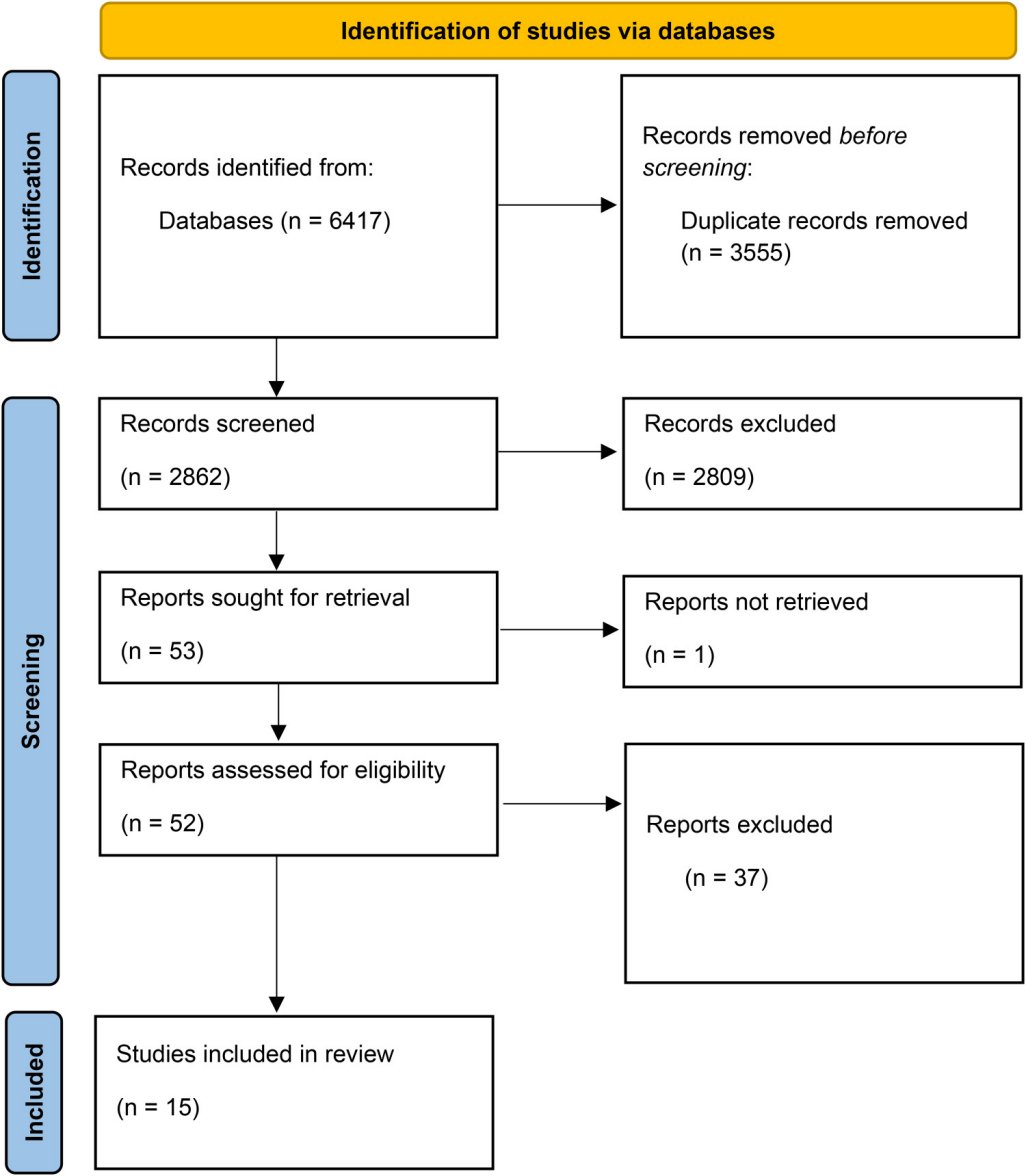
Flowchart.

The selection process is illustrated in the PRISMA^40^ flowchart (Figure 1). The search yielded 6417 articles. Duplicates (*n* = 3555) were removed using Zotero 6.0 software.^41^ The remaining 2862 abstracts were screened manually in Rayyan software,^42^ applying the inclusion and exclusion criteria. The artificial intelligence agent within software was not used. Each abstract was independently assessed by two researchers (MP and A-CB), with discrepancies or “Maybe” classifications resolved through discussion. This process excluded 2809 studies.

In the next stage, 53 full-text articles were retrieved for eligibility assessment. Of these, 37 were excluded due to lack of relevance, and one could not be located, resulting in 15 studies included in the final review.

### Thematic analysis

Finally, we conducted a reflexive thematic analysis of the 15 included studies.^43^ The researchers (MP, A-CB, and ET) read through the articles and coded specific emotional expressions. The specific emotional expressions were clustered into categories based on emotional similarity^44^ and refined through iterative team discussions to ensure conceptual clarity and alignment with Scheff's^22^ theory of social bonds. Following principles of reflexive analysis, we emphasized the interpretative role of the researchers and maintained reflexivity through continuous dialogue and revisiting of the dataset.^45^ This process generated six overarching categories of emotions (see Table 1) which were thoroughly discussed and refined to ensure they were mutually exclusive and devoid of overlapping meanings.

**Table 1.** Coding emotional expressions into categories.

Emotional expressions in the reviewed research papers	Categories of emotions
Satisfaction, ease, relaxation, peace, contentment, calmness	Comfort
Dissatisfaction, bewilderment, indifference, detachment	Discomfort
Happiness, enthusiasm, connectedness, achievement, hope, pride	Joy
Agitation, disappointment, discouragement, annoyance	Anger
Confidence, openness, trust, feeling valued	Safety
Insecurity, fear, anxiety, sadness, embarrassment, loneliness, distress	Vulnerability

## Findings: emotional expressions and their indications of the quality of social bonds

This section explores how emotional expressions emerge in the interactions between care workers and residents with dementia when digital technologies are used. We begin by summarizing the research papers included in the review (see Table 2), followed by a detailed discussion of the findings.

**Table 2.** Summary of the research papers included in the review.

Author(s), year, country	Aim of the study	Method and data	Technology	Emotions	Impact on professional relationship(s)
Blindheim et al., 2023, Norway^46^	Explore how residents and care workers experience interacting with a social robot	Observations of interventions (20 sessions) including residents (4–6) and care workers (1–3) per session. Also, interviews with residents (3) and care workers (4)	Semihumanoid robot: Pepper	Joy, satisfaction, humor, connectedness, confidence, pride, worry, frustration, insecurity, fear, lack of confidence	Increase of communal activities, social stimuli, joint interaction, and communication; humor and “joking relationships.” Critical voices about care ethics regarding dignity, and emotions of disappointment when realizing the limitations of the robot
Dove and Astell, 2019, Canada^47^	Explore how motion-based technology is used as group activity involving people with cognitive impairment and care workers	Observations (20) of game play interventions including people with cognitive impairment (23), and care workers (2)	Motion-based technology: Xbox Kinect	Joy, enthusiasm, trust, social atmosphere, connectedness, laughter	Positive contribution to social interaction, in both embodied and verbal ways; doing things together
Goodall et al., 2021, Norway and Portugal^48^	Create a model of how identity and relationships are promoted through technology	Interviews with residents (7) and care workers/relatives (13) about experiences of intervention	Multisensory stimuli room: Sense-garden	Happiness, enthusiasm, joy, comfort, respect, safety, dissatisfaction, sadness, anger	Openness and vulnerability when sharing memories, and experiences, with others; improved understanding of the resident, which provided benefits for the relationship (s.14); sense of connection; feeling of transportation and energy
Goodall et al., 2021, Norway, Belgium, Portugal, and Romania^49^	Explore care workers’ experiences when using multisensory stimuli technology	Observations and interviews with care workers (8) when using technology in sessions with residents	Multisensory stimuli room: Sense-garden	Pride, joy, enthusiasm, hope, meaningfulness, humor, frustration, disappointment, sadness, bewilderment, anger	Build and foster relationships, e.g. by learning residents’ individual preferences and history; enhanced social connection through meaningful activities
Gustafsson et al., 2015, Sweden^50^	Explore the reactions of residents (4), and experiences of care workers (11) when using robot technology	Mixed-methods design with quantitative single-case observations and qualitative interviews	Robot animal: JustoCat	Relaxation, joy, pride, peace, comfort, meaningfulness, curiosity, worry, loneliness, agitation	Opportunity for emotional release; enabling the expression of positive emotions toward the robot; engaging in humor and play; prompting social interaction between residents and care workers
Hollinda et al., 2023, Canada^51^	Explore the elements of facilitating digital storytelling	Qualitative study involving residents (19) and care workers (5)	Multimedia sources, such as audio, video, and text	Comfort, trust, ease, confidence, meaningfulness, satisfaction, achievement	Collaborative relations using technology to co-create identity stories
Hung et al., 2018, Canada^52^	Explore feasibility and acceptability of a tablet technology	Mixed-methods design involving video recorded observations (200 min) of residents (4) and interviews with care workers (2)	Tablet computer with personalized video content from relatives	Calmness, safety, comfort, frustration, anger, fear, anxiety, disappointment	Care workers perceived the tablet as a useful resource to engage family members and contribute to their relationships with residents
Lazar et al., 2016, USA^53^	Explore feasibility and acceptability of a multipurpose technological system	Mixed-methods design involving observations of sessions with residents (5), care workers (7), and relatives (4). Also, interviews were conducted with care workers	Computer system designed for older adults with cognitive impairment (hardware and software)	Enjoyment, happiness, joy, pride, anticipation, frustration, fear, bewilderment	Informed care workers about the residents’ likes and dislikes: involvement of facilitator (care worker) was key to positive experiences/emotions.
Purves et al., 2015, Canada^54^	Explore and pilot test a regionally adapted version of a digital conversation aid	Focus group interviews with residents (39), and video recorded interventions with residents (3) and care worker (1)	Touch screen computer with multimedia content: CIRCA	Joy, enthusiasm, frustration, sadness	Computer-mediated interaction contributes to meaningful relationships and deepened understanding of individuals’ histories
Samuelsson and Ekström, 2019, Sweden^55^	Explore how digital communication support can be used in interaction	Video recorded interactions (29) and interviews with residents (3) and care workers (3)	Tablet computer with multimedia content: CIRCA/CIRCUS	Amusement, Joy, ease, humor, curiosity, frustration, distress, sadness	New knowledge about residents’ histories; less demanding responsibility for the care worker, since residents become more active in interaction
Smith et al., 2020, UK^56^	Explore how tablet computers can be incorporated in activities at a care home	Video recorded observations (16 h) of group sessions with residents (12) and care workers (8)	Tablet computers with various apps, such as games, trivia, music, camera/video	Joy, humor, awareness, confidence, indifference, detachment	Focusing on activities that can promote enjoyment and emotions of achievement and self-worth can enhance individual well-being as well as meaningful co-operative activities
Swan et al., 2018, Australia^57^	Explore care workers and residents’ experiences of using tablets	Mixed-methods design involving surveys and interviews with care workers (8), and interviews with residents (7)	Tablet computers	Joy, awe, comfort, sense of mastery, satisfaction, anxiety, fear, annoyance	Enhanced social connection between residents and care workers, who experienced the connection as satisfying and as a motivation to be more proactive in their relationships with residents
Tanioka et al., 2021, Japan^58^	Examine residents’ experience of interaction with robotics and care workers	Video recorded interactions with residents (2) and care workers (2)	Humanoid robots: Pepper and Kabochan	Joy, meaningfulness, empathy, safety, comfort, confidence,	Transactive engagements with robots can facilitate the experience of joy
Tobiasson et al., 2015, Sweden^59^	Explore residents’ experience of using exercise games	Participatory case study design (5) involving (video) observations and interviews with residents (22) and care workers (not specified)	Digital games: Nintendo Wii Sports	Joy, humor, connectedness, confidence, achievement, frustration, embarrassment, ambiguity	Enhanced social connection both verbally and physically by playing games together; games provided the organization with new work routines; interactions changed the relationship, making it symmetrical
Whelan et al., 2020, Ireland^60^	Explore how social robots affect the resilience of residents	Multiple case study design involving observations and interviews with residents (19), care workers (6), and relatives (7)	Social robot: Mario	Joy, happiness, satisfaction, confidence, meaningfulness, disinterest, unhappiness, distress	Co-creation of meaningful activities contributed to the relationship, e.g. the care workers learned residents’ preferences and likes

Although the review applied broad and nonrestrictive criteria regarding digital technology, four categories of technologies dominate the final selection of full papers: (a) robots (e.g. Mario, Pepper, and JustoCat), (b) game consoles (e.g. Xbox, Nintendo, and Wii), (c) tablet computers (e.g. iPads with various multimedia applications), and (d) virtual environments (e.g. Sense-garden). As the analysis will show, these technologies are engaged in diverse ways, eliciting a range of emotional expressions—from joy and curiosity to frustration and indifference. These emotions, in turn, provide insight into the varying qualities of social bonds formed or disrupted through technology-mediated interactions.

Instead of categorizing the findings by type of technology, we organize them according to contrasting emotional expressions, to more closely reflect the study's central aim: exploring how the (dis)affordances of digital technologies shape the quality of social bonds in dementia care. Emotions are not only outcomes of these interactions but also key indicators of relational quality—they reveal how residents and care workers experience each other through technology. By foregrounding emotional expressions, this structure allows for a more nuanced and person-centered account of how different technologies either support or challenge emotional attunement. In this way, technology is treated not as the main focus, but as a mediator of relational experience.

### Comfort versus discomfort

In the reviewed literature, comfort is a commonly observed emotion in situations where digital technology is approached by care workers and residents in relaxing and calming ways. The articles usually treat comfort as a pleasant state or a human being in a relaxed relationship with their environment, while discomfort is seen as its opposite, as an individual having an unpleasant reaction to their environment. When viewed through the lens of (dis)affordance theory, these emotional outcomes can be understood not simply as internal states, but as emergent properties of the interactional possibilities—or limitations—that technologies create within care environments.

Digital technologies such as tablets afford specific forms of interaction that facilitate emotional attunement. Several studies demonstrate how a range of interactional and calming activities on tablet computers can facilitate meaningful engagements between care workers and residents.^52,56,57^ For instance, Purves et al.^54^ describe how music applications afford shared conversations about artists and songs, as well as sparking cheerful moments of singing together. Similarly, Lazar et al.^53^^(p379)^ find that playing music that both residents and staff liked “bridges the age gap and the generation difference,” and leads to “a sense of connection.”

Hollinda et al.^51^^(p857)^ further illustrate how care workers engage in digital storytelling platforms to share emotions, “such as sharing sadness about a loss or laughter in a funny story,” and through relating, care workers “created a comfortable environment.” Here, comfort arises not from technology per se, but from the triadic interaction between care workers, residents and digital technology. Thus, the storytelling platform enables care workers and residents to create conditions under which emotional coherence with residents becomes possible.

In different ways, these papers show how digital platforms support opportunities for mutual recognition and satisfaction, thereby supporting Scheff's notion of emotional attunement and secure social bonds. Samuelsson and Ekström^55^ demonstrate that viewing photographs together using multimedia applications resulted in more active and enjoyable verbal interactions than those without technological support. Staff felt that having a tablet made their communication with the residents easier, because the interaction felt less demanding. Without the tablet's support, care workers perceived their interactions with residents as repetitive, as talking about the same things over and over. The literature suggests that interactions mediated by digital technologies can contribute to an enhanced degree of emotional coherence between care workers and residents, that is, it promotes secure social bonds.

Virtual environments similarly afford immersive, shared attention. Goodall et al.^49^ describe how care workers and residents engaged in moments of joint focus when sitting together and talking in a room that has been turned into a space for visual multimedia content, creating a calm and tranquil atmosphere. The affordances of the room—the absence of distractions, the visual immersion—enabled a relational space conducive to shared emotional experiences of comfort and pleasure that indicate stable social bonds between the participants.

The literature also reveals that social robots can be approached in ways that generate emotions of comfort and facilitate social bonds. For instance, both Blindheim et al.^46^ and Whelan et al.^60^ demonstrate that robots can facilitate peaceful verbal interaction about residents’ personal interests, for example about plants, music, or other memories. Gustafsson et al.^50^ detail how a robot cat was approached as a medium to spur interaction between care workers and residents. For example, the robot cat prompt heartful talk about previously owned real cats and helped residents recollect their amiable memories of the places and events they associated with cats and animals. The positive emotions and expressions of contentment which these interactions provoked, therefore, shows that robots can generate opportunities for co-constructing meaning, reinforcing secure socials bonds through shared histories and emotional recognition.

Yet, digital technologies can also hinder emotional attunement, leading to feelings of discomfort. The virtual environments which Goodall et al.^49^ observe that disturbances in the virtual environment—such as additional participants disrupting personal storytelling—led to confusion, indifference, and emotions of discomfort among residents. Here, the participants’ engagement with the technology's possibility for a shared narrative space was compromised, generating emotional dissonance.

Similarly, Lazar et al.^53^ and Smith et al.^56^ note that narrative continuity was fragile, requiring care workers to re-establish context regularly. The residents needed information repeated to them both in each session and between the sessions.^53^^(p382)^ To create a shared moment of “let's try this together” in a technology session,^56^^(p1599)^ and to avoid misunderstandings and feelings of indifference, the care workers had to remind residents what had happened last time. The failure to do so led to emotional discomfort, a sign of insecure social bonds.

The findings suggest that when digital technologies serve as interactional resources for storytelling and shared narratives, it can evoke emotions of comfort and rest. By providing a medium for mutual verbal recognition, technology can be approached by care workers and residents to affirm each other's personal stories and narrated sense of self. From Scheff's perspective, this can be understood as an instance where technology fosters attunement between participants. Conversely, when technology results in a lack of shared information or disrupts narrative continuity between care workers and residents, it generates emotional discomfort, indicating insecure social bonds. Drawing on Scheff's theory, we interpret these disruptions as a breakdown in attunement, where participants fail to recognize and validate each other's narrated sense of self.

Following Scheff's theory of social bonds, we find that emotions of comfort and discomfort are primarily associated with the secure or insecure qualities of the social relation between care worker and resident. Moreover, we find that these emotional expressions relate to *verbal (dis)affordances*. By maintaining continuity in shared narratives through technology-mediated interactions, technology is engaged by care workers as a resource to strengthen rather than weaken social bonds with residents. Finally, it is notable that game consoles, although part of the technological repertoire, were not associated with comfort or discomfort. Rather, these devices were linked to expressions of joy, suggesting they may afford a different relational dynamic altogether. This distinct affective register will be discussed in the following section.

### Joy versus anger

Emotional expressions of joy—particularly those emerging from play and humor—are a recurring theme in studies involving care workers and dementia care residents’ engagement with game consoles and digital games on tablets.

For instance, Tobiasson et al.^59^ report many instances of fun and laughter during Wii training sessions with dementia care residents. The care workers supported the residents to move their hands and arms as the game required. The situation consisted of both gamers (residents, care workers) and audience (other residents). By cheering, teasing, coaching, and competing, the setting provided an arena for mutual sharing of joyful and happy emotions, for both participants and spectators. Tobiasson et al.^59^ conclude that both the cheering and the teasing seemed to have a positive impact on the atmosphere and that both could be understood as supporting the creation of secure social bonds between the participants. Similarly, Smith et al.^56^^(p1598)^ note that tablet computers encouraged joyful interactions by providing “opportunities for increased conversation and laughter.” Both Tobiasson et al.^59^ and Smith et al.^56^ emphasize the role of embodied action, illustrating that physical gestures, when coupled with verbal interaction, are central to shared moments of happiness enabled by the relational engagement with game consoles.

The combination of verbal and physical gestures as contributing to the emotional content of the technology-mediated interaction between care workers and residents is even more evident in the studies that involve robots. Regardless of form—whether humanoid^58,60^ or animal-like^50^—robots are designed to inspire physical interaction and sensory stimulation in a way that tablet computers or virtual environments could not. For instance, robot animals, such as cats or dogs, are objects designed to be touched and cuddled but also talked about and with. Gustafsson et al.^50^^(p52)^ stress that the robot cat was perceived as “something extra special,” fostering expressions of pride, joy, and mutual connectedness among residents and care workers. The robot's affordances—its softness, its simulated purring, its familiar form—elicited affectionate behavior, transforming it into a relational object that facilitated the sharing of bright emotions and personal narratives.

Although humanoid robots may not provoke the desire to cuddle them, as Blindheim et al.^46^^(p187)^ have shown, their physical appearance can prompt a desire for emotional interaction:

The robot's embodiment also afforded a different use of humour. They [the residents and care workers] could tell jokes about Pepper [the humanoid robot], and these small, joyful remarks were a source of positivity and laughter among residents and healthcare professionals, bringing the participants together as a group.

Laughter and so-called “joking relationships” were a salient feature of these encounters. Pepper's presence supported a sense of community based around the fact that the participants were doing something together.^46^^(p190)^ Similarly, Whelan et al.^60^ who observe that care workers and residents took a “shared delight” in their interactions with Mario, a humanoid robot. Such findings of emotional expressions of joy are clear indications of how encounters with digital technology can establish secure social bonds between the participants.

Some of the reviewed papers show how, as with all technologies, things can break down, malfunction, or simply demand a lot of manual labor and time because of a low level of autonomy. The time spent tinkering with technology to get it to work, unsurprisingly, can cause friction in the interactions between care workers and residents. This is one of the most common reasons stated in the reviewed literature for the demonstration of emotions of frustration and anger among residents as well as care workers.^52,53,59^

For instance, Blindheim et al.^46^ show that the robot's low level of autonomy made the care workers experience the interactions with it as something that had to be continuously maintained and mediated. The care workers had expected the robot to “free up hands, which it did not.” Instead, “[S]omebody had had to control him [the robot], which led to us spending more time initiating and controlling the robot than we would have if I had used myself for another activity.” In this example, the care workers felt disappointed when the technology did not free up their time, or make their interactions with residents any easier, but on the contrary ended up acting as a “time sink.”^46^^(p187)^ Moreover, the residents expressed frustration and disappointment when they were physically unable to follow robotic exercise programs. In sum, Blindheim et al.'s study reveals the expression of emotions of disappointment and frustration by care workers and residents when they are confronted with digital technologies that have limited capacity for action, or technology that reveals their own physical or mental limitations. In these cases, technology became a site of interactional failure, undermining the relational dynamics and contributing to a low degree of harmony in the relationships between care workers and residents, that is, of insecure social bonds between them.

The emotional expressions of joy and anger by care workers and residents when engaging in verbal and physical joint activities with technology can be interpreted as indications of a high or low degree of emotional attunement in their relationships. These expressions reflect varying degrees of emotional coherence, which, in this context, are precariously shaped by what technologies afford—or disafford—in terms of joint activity and embodied participation. These findings underscore that the technologies do not simply involve *verbal affordances*—by engaging participants in joint communication—but also include *embodied affordances* and sensory dimensions of social interaction. Playful, recreational engagements afford shared movement and coordinated physical actions, which serve as mechanisms for reinforcing social bonds. However, if the technology fails to facilitate a co-constructive moment, does not function properly, or falls short of expectations, it may also evoke emotions of disappointment and annoyance denoting the insecure quality of the social bond. In such cases, emotional attunement between participants is compromised, leading to a sense of disconnection.

Finally, virtual environments appear to operate on a distinct emotional register. Unlike gaming consoles or robots, virtual rooms primarily afford emotions of comfort or discomfort—as discussed above—rather than joy or frustration. This differentiation further emphasizes the importance of situating technological engagement within the specific affordances each device or system makes available to participants.

### Safety versus vulnerability

The third category of emotional expression emerging from care workers’ and residents’ engagement with digital technology pertains to social situations marked by vulnerability and exposure. Regarding the use of virtual environments, Goodall et al.^48^ describe how residents spoke about personal pictures and videos from the past. These digital encounters sometimes involve images of deceased family members, evoking complex emotional states such as nostalgia, sorrow, and sadness. Goodall et al.^48^^(p13)^ conclude that “[t]he openness inside the virtual environment seems to prompt a level of vulnerability amongst participants, which allows for a free expression of emotions, without fear of judgement.” This setting, then, functioned as a social space of safety, in which vulnerability could be disclosed and received empathetically—thus fostering emotional coherence between participants. Similar experiences can be seen in the studies involving tablet computers. Hollinda et al.^51^ observe situations where tablets were used by care workers and residents to facilitate co-produced storytelling. These situations were sometimes marked by emotion of confidence but also included the emotion of vulnerability. As Hollinda et al.^51^^(p859)^ state:

[I]t may be difficult for people to open up to strangers. In the digital storytelling process, participants are required to open up to the facilitators [care workers]. A degree of trust was developed when facilitators [care workers] built relationships with and related to participants over sessions.

In this example, because the virtual environment provided a context for the residents to reveal their vulnerability and because the care workers displayed sensitivity to this and responded with empathy, emotions of confidence reflecting strong social bonds were developed over time. Hollinda et al.^51^ argue that emotions of trust are heavily dependent on care workers’ competence to support residents’ capacity to be actively involved in decision-making processes and in technology-mediated interactions as co-creators, rather than passive recipients. If care workers had instead exercised unilateral control over the digital interaction, it would have constituted a disaffordance, undermining residents’ agency and potentially evoking emotions of helplessness or incompetence.

These findings suggest that emotions of safety arise when technologies are engaged in ways that acknowledge and respond to the vulnerability of the participants. Indeed, embodied expressions of care—facilitated by digital tools—can deepen trust. Dove and Astell,^47^ in their study of game console use, observe how care workers physically supported residents with mobility impairments. By standing beside them, demonstrating required movements, and offering tactile guidance (e.g. placing their hand over the resident's), care workers supported embodied participation in ways that protected residents from feeling belittled or excluded.^59^ This support not only provided the residents with an opportunity to take part in a gaming activity—and have fun—but, as Dove and Astell,^47^^(p2197)^ point out, it also appeared “to establish a level of trust between the trainer and the client.”

Encounters with robots also appear to facilitate embodied interactions.^46^ For instance, Gustafsson et al.^50^ observe how care workers were able to draw on a robot cat as a material resource for providing a feeling of security. They note that, by placing the cat on a resident's chest or lap and encouraging them to interact with the cat by sitting next to them and petting the cat, the care worker could help the resident relax when anxious or upset.

The above examples illustrate how, through encounters with technology, care workers manage to address residents’ insecurities and fears in an empathic manner and, through joint digital activities, together work to manage residents’ emotional or physical vulnerabilities. By responding to these vulnerabilities with attuned support—both verbal and embodied—care workers create the conditions for secure and harmonious social bonds. However, these bonds are precarious, as technological breakdowns, mismatched expectations, or emotional misattunement can produce the opposite effect.

For example, Hung et al.^52^ describe how some residents responded to tablet computers with aggression and fear, pushing care workers away, yelling, or physically resisting engagement. These reactions were often tied to misunderstandings or discomfort with the technology, interpreted by residents as invasive or threatening. As one care workers states: “I see our patients hit and kick, sometimes yell at us, push or shoo us away. Maybe they misinterpret what we are doing and do not trust us or know we are trying to help.”^52^^(p356f)^ Such interactions suggest a disaffordance of safety, where tablet computers become a source of fear or anxiety rather than support.

Crucially, emotional vulnerability is not limited to residents alone. Care workers, too, may experience fear, uncertainty, or a lack of competence when engaging with unfamiliar technologies. Blindheim et al.^46^ recount the story of a care worker who brought her son to work to assist with operating a social robot. Her anxiety about making mistakes and being perceived as unqualified constituted a form of professional vulnerability—an emotional hindrance to fully engage with residents with the help of the robot. Similarly, Swan et al.^57^ report that both residents and staff felt pressure to use tablets “properly,” with some residents expressing age-related discomfort: “I’m too old for that.” These findings reveal that emotional safety in digital encounters is mutually dependent; when care workers themselves do not feel confident or supported, they are less able to protect the emotional integrity of their interactions with residents.

Viewed through the lens of Scheff's theory of emotional attunement, these instances of vulnerability—whether acknowledged or denied, managed or neglected—signal the relative strength or fragility of the social bond. When both care workers and residents feel emotionally safe and seen in their joint interactions with technology, a secure bond is established. When fear, uncertainty, or lack of mutual recognition predominates, this bond is compromised. Digital technology introduces a potential source of disruption, as uncertainty in its application can engender feelings of doubt or inadequacy, particularly among those who struggle to adapt to new tools. The emotional fragility arising from technological uncertainty highlights the role of *ethical affordances* in technology-mediated interaction, where both care workers and residents must feel supported in their engagement with digital resources.

In sum, emotions of safety and vulnerability in technology-mediated care interactions provide a window into the relational quality of the care relationship. Technologies that afford possibilities for empathetic, embodied, and co-constructive interaction help reinforce emotional attunement and secure social bonds. Those that disafford mutual understanding or exacerbate fears and insecurities undermine the care relationship, revealing its fragility. The ethical imperative, therefore, is not merely to introduce digital technologies into care but to support context-sensitive and responsive interaction, where both care workers and residents are supported in their engagement with digital technologies.

## Discussion: technology's impact on social bonds

This review has explored how emotional expressions in technology-mediated interactions shape social bonds between care workers and residents with dementia. By integrating Scheff's theory of social bonds with affordance theory, we demonstrated that digital technologies are not neutral tools but active mediators of relational quality. Secure bonds were associated with emotions of comfort, joy, and safety, whereas discomfort, frustration, and vulnerability signaled relational insecurity. These emotional dynamics arise both from interpersonal exchanges and from the affordances and constraints embedded in technological design.

Affordance theory highlights how technologies enable or hinder joint action, highlighting their constitutive role in care interactions. Consistent with prior research on the relational dimensions of care technologies,^17,61^ our analysis shows that technologies shape the emotional texture of care. For instance, audio-visual applications offer possibilities for verbal interaction, such as conversation, storytelling, and the sharing of personal histories, which supports emotions of comfort and a coherent sense of self. Game consoles offer embodied affordances that elicit joy through shared physical engagement and coordinated movement. Social robots afford possibilities for empathetic and safe-guarding interaction that help protect the vulnerability of the residents, thus, reinforcing emotional attunement and secure social bonds.

Conversely, our analysis also highlights the risks of emotional dissonance. Disrupted storytelling, technological breakdowns, or insufficient protection of residents’ vulnerabilities can trigger frustration, discomfort, or shame—emotions that undermine social bonds. These observations resonate with critiques of care technologies that emphasize their potential to alienate or disempower residents when poorly integrated into relational practice.^62^

Taken together, these findings extend Scheff's binary of pride and shame by identifying three types of affordances—verbal, embodied, and ethical—that are closely linked to emotional expressions in care. While Scheff's theory did not account for sociotechnical mediation, our synthesis suggests that these affordances provide a valuable lens for understanding how technologies actively shape relational quality. We found that while most digital technologies offer verbal and ethical affordances, embodied affordances were largely absent from virtual systems, indicating that the design of digital tools matters profoundly for the kinds of emotions and bonds they elicit.

From a broader perspective, this review positions social bonds in dementia care as sociotechnical constructs. Emotions should not be understood as private psychological states, but as emergent features of a relational ecology in which technologies enable, constrain, and reconfigure possibilities for emotional attunement.^63^ Recognizing this allows for a more precise analysis of how technologies may support or undermine relational quality in care settings.

This study underscores the need to approach care technologies not only as tools for efficiency but also as mediators of emotional and social dynamics in dementia care. By foregrounding the relational and emotional dimensions of technology use, it highlights the potential of designing and implementing systems that are responsive to the lived experiences of both residents and care workers. At the same time, critical perspectives on the dehumanizing and isolating effects of such technologies^10,11^ highlight the risks of neglecting these dimensions.

In an era of increasing digitalization and pressure toward cost-effectiveness, measurable outcomes and productivity often take precedence over less quantifiable qualities such as empathy, presence, and interpersonal connection. Importantly, the role of technology in dementia care differs from its role in strictly medical contexts, such as acute hospital settings, where the primary aims involve maximizing autonomy, functional independence, and biomedical outcomes. Dementia care, by contrast, is grounded in relationality, interdependence, and trust, reflecting the progressive decline in residents’ cognitive and communicative abilities. Future developments in this area must therefore ensure that technological innovations reinforce, rather than undermine, these essential foundations of care.

### Implications

The findings of this review highlight the importance of developing and supporting *relational competence* in dementia care as care environments become increasingly digitized. Care workers’ ability to balance technological affordances with embodied, verbal, and ethical dimensions of interaction will likely play a decisive role in maintaining and strengthening social bonds with residents. Training programs and organizational policies should therefore not only focus on technical proficiency but also explicitly address how to adapt communication strategies, manage breakdowns in technological systems, and remain attentive to residents’ emotional needs. By doing so, digitalization can be integrated into care practices in ways that enhance, rather than disrupt, the quality of relationships.

At the same time, the review underscores the need to broaden the scope of inquiry beyond technologies explicitly designed for relational purposes. Background technologies such as surveillance systems, automated alarms, or medicine dispensers may reconfigure the rhythms of everyday care and influence opportunities for human contact. Their potential to supplement—or replace—moments of interpersonal interaction raises critical questions for the future of dementia care. Addressing these questions requires attention not only to the efficiency and safety such technologies provide, but also to their subtle effects on social connectedness and emotional well-being.

### Limitations

While this narrative review offers insights into how digital technologies shape emotional and relational dynamics in dementia care, certain methodological limitations should be noted.

Thematic analysis is typically used with primary data, and its application to secondary sources like published literature has been debated.^64,65^ Nonetheless, we argue it suits our explorative aim, enabling theory-informed interpretations (sometimes obvious, sometimes subtle) across diverse studies—though it inevitably involves some subjectivity.

Moreover, narrative reviews have been criticized for a lack of methodological clarification and rigor.^38^ To mitigate this, we applied a transparent and rigorous process in our literature search, including clear inclusion/exclusion criteria and quality control measures. Our account of the review process demonstrates our conscientious efforts to pursue a robust and impartial methodological approach.

## Conclusions

This review demonstrates that digital technologies are not neutral tools but active participants in shaping the emotional and relational dynamics of dementia care. By integrating affordance theory with Scheff's framework of pride and shame, we showed how verbal, embodied, and ethical affordances can either reinforce or strain social bonds, positioning emotional expressions as key indicators of relational security. These insights highlight the need to strengthen care workers’ relational competence—balancing technological proficiency with communication skills, ethical sensitivity, and emotional attunement—so that digitalization enhances rather than disrupts caregiving. At the same time, attention must extend beyond technologies designed for interaction to include background systems such as surveillance or automated dispensers, whose subtle restructuring of everyday routines may influence opportunities for human connection. Sustained research and practice are therefore essential to ensure that technological innovation in dementia care supports, rather than undermines, the quality of social bonds.

## Appendix: Complete search string

[Table table3-20552076251411208]

**Table table3-20552076251411208:**

Search area	Search term
Population: care workers and persons with degenerative disorders	(Dementia[MeSH] OR dement*[tiab] OR senile[tiab] OR “memory disorder*"[tiab] OR Alzheimer disease[MeSH] OR Alzheimer*[tiab]) AND (((“Health personnel"[MeSH] OR “health personnel"[tiab] OR “care provider*"[tiab] OR “healthcare provider*"[tiab] OR “care worker*"[tiab] OR “healthcare worker*"[tiab] OR “health worker*"[tiab] OR healthworker[tiab] OR “health professional*"[tiab] OR “care professional*"[tiab] OR “healthcare professional*"[tiab] OR Careworker*[tiab] OR “Professional caregiver*"[tiab] OR Physicians[MeSH] OR physician*[tiab] OR Doctor*[tiab] OR Clinician*[tiab] OR Practitioner*[tiab] OR Nurses[MeSH] OR nurse*[tiab] OR nursing[MeSH] OR nursing[tiab] OR “Occupational therapists"[MeSH] OR “occupational therapist*"[tiab] OR “Physical therapists"[MeSH] OR “Physical therapist*"[tiab] OR Physiotherapist*[tiab] OR “Speech therapy"[MeSH] OR “speech therapist*"[tiab] OR “Speech-Language Pathology”[MeSH] OR “Speech-Language Patholog*”[tiab] OR Audiologists[MeSH] OR audiologist*[tiab] OR pharmacists[MeSH] OR pharmacist*[tiab] OR nutritionists[MeSH] OR nutritionist*[tiab] OR institution*[tiab])
Context *a*: relations between population when using technology	(“social behavior”[MeSH] OR “Social interaction”[MeSH] OR “Social structure”[MeSH] OR “Social isolation”[MeSH] OR “Social cohesion”[MeSH] OR “Social skills”[MeSH] OR “social networking”[MeSH] OR social[tiab] OR sociability[tiab] OR socio-technical[tiab] OR “Group dynamics”[MeSH] OR “Group dynamics”[tiab] OR “team dynamics”[tiab] OR “group structure”[MeSH] OR “group structure*”[tiab] OR “team structure*”[tiab] OR Reciprocity[tiab] OR interact*[tiab] OR engag*[tiab] OR participat*[tiab] OR relation*[tiab] OR interperson*[tiab] OR joint[tiab] OR communication[MeSH] OR communicat*[tiab] OR narration[tiab] OR conversation[tiab] OR intima*[tiab] OR familiar*[tiab] OR transaction*[tiab] OR connection*[tiab] OR intersubjectivity[tiab] OR role-taking[tiab] OR interrelate*[tiab] OR isolat*[tiab] OR withdraw*[tiab] OR unsocial*[tiab] OR asocial*[tiab] OR presocial[tiab] OR pre-social[tiab] OR contact[tiab] OR company[tiab] OR dyad[tiab] OR triad[tiab])) OR (“Professional-Patient Relations"[MeSH] OR “Physician-Patient Relations"[MeSH] OR “Nurse-Patient Relations"[MeSH] OR “Interpersonal relations"[MeSH] OR “professional-patient*”[tiab] OR “provider-patient*”[tiab] OR “therapeutic alliance”[tiab] OR “dementia care”[tiab]))
Context *b*: digital technology	(“digital technology"[MeSH] OR “digital technolog*"[tiab] OR “information technology"[MeSH] OR “information technolog*"[tiab] OR “information and communication technolog*”[tiab] OR ICT[tiab] OR “assistive technolog*”[tiab] OR robotics[MeSH] OR robot*[tiab] OR internet[MeSH] OR “internet-based intervention”[MeSH] OR internet*[tiab] OR “mobile applications"[MeSH] OR “mobile application*"[tiab] OR “phone application*"[tiab] OR app[tiab] OR apps[tiab] OR “artificial intelligence"[MeSH] OR “artificial intelligence"[tiab] OR AI[tiab] OR software[MeSH] OR software[tiab] OR hardware[tiab] OR telephone*[tiab] OR “cell phone"[MeSH] OR “cell phone*"[tiab] OR smartphone[MeSH] OR smartphone*[tiab] OR “mobile phone*"[tiab] OR smartwatch*[tiab] OR ipad*[tiab] OR iphone*[tiab] OR tablets[tiab] OR ios[tiab] OR android[tiab] OR touchscreen*[tiab] OR touch-screen[tiab] OR “text messaging"[MeSH] OR “text messag*"[tiab] OR “short messag*"[tiab] OR “virtual reality"[MeSH] OR “virtual realit*"[tiab] OR “virtual room*”[tiab] OR “augmented reality”[tiab] OR VR[tiab] OR CRDL[tiab] OR computers[MeSH] OR computer*[tiab] OR “video games"[MeSH] OR games[tiab] OR gaming[tiab] OR gamification*[tiab] OR gamification[MeSH] OR exergaming[MeSH] OR exergam*[tiab] OR Nintendo[tiab] OR Wii[tiab] OR telemedicine[MeSH] OR telemedicine[tiab] OR telehealth[tiab] OR tele-health[tiab] OR telecare[tiab] OR tele-care[tiab] OR telepresence[tiab] OR tele-presence[tiab] OR mhealth[tiab] OR ehealth[tiab] OR m-health[tiab] OR e-health[tiab] OR gerontechnolog*[tiab])
Concept: emotions, experiences, attitudes	(emotions[MeSH] OR Emotion*[tiab] OR Regret*[tiab] OR Feel*[tiab] OR Mood[tiab] OR sense[tiab] OR Affects[MeSH] OR affect*[tiab] OR Aggressi*[tiab] OR Anger[tiab] OR angry[tiab] OR Rage[tiab] OR Anxiety[tiab] OR anxious[tiab] OR nervous*[tiab] OR Apath*[tiab] OR Bereave*[tiab] OR Calm[tiab] OR Grief[tiab] OR grievous[tiab] OR Bored*[tiab] OR Depress*[tiab] OR Disgust*[tiab] OR Euphori*[tiab] OR Fear*[tiab] OR Panic[tiab] OR Forgiv*[tiab] OR Frustrat*[tiab] OR Guilt*[tiab] OR Shame*[tiab] OR Happiness[tiab] OR happy[tiab] OR enjoy*[tiab] OR Hate*[tiab] OR Hope*[tiab] OR hopeful[tiab] OR vital[tiab] OR Hostil*[tiab] OR Jealous*[tiab] OR Loneliness[tiab] OR lonely[tiab] OR Love[tiab] OR loving[tiab] OR Pleasure[tiab] OR Distress[tiab] OR Sadness[tiab] OR sad[tiab] OR sorrow*[tiab] OR painful[tiab] OR Satisfaction[tiab] OR satisfied[tiab] OR Stress*[tiab] OR well-being[tiab] OR delusion*[tiab] OR fatigue[tiab] OR tired*[tiab] OR optimis*[tiab] OR pessimis*[tiab] OR empath*[tiab] OR sympath*[tiab] OR sensiti*[tiab] OR compassion*[tiab] OR together*[tiab] OR meaningful*[tiab] OR attachment[tiab] OR belonging[tiab] OR attitude*[tiab] OR “attitude to computers"[MeSH] OR experiences[tiab] OR perspective*[tiab])
